# Human epidermal growth factor receptor 2-positive microinvasive breast carcinoma with a highly aggressive course: a case report

**DOI:** 10.1186/1756-0500-7-325

**Published:** 2014-05-31

**Authors:** Cvetka Grasic Kuhar, Erika Matos

**Affiliations:** 1 Department of Medical Oncology, Institute of Oncology Ljubljana, Zaloska 2, SI-1000 Ljubljana, Slovenia

**Keywords:** Breast cancer, Microinvasion, Ductal carcinoma in situ, Therapy

## Abstract

**Background:**

Microinvasive ductal carcinoma in situ of the breast is a rare entity defined as ductal carcinoma in situ with invasive foci measuring no more than 1 mm. In general, the outcome is excellent, similar to ductal carcinoma in situ. We report a patient with breast ductal carcinoma in situ with microinvasion who died eight months after diagnosis due to progression of the disease – liver metastases. This is the first report in the literature of such an aggressive course.

**Case presentation:**

A 47-year-old Caucasian woman presented with mammographic-detected suspicious microcalcinations in an area of 8.6 x 6 cm. A radical mastectomy with a sentinel lymph node biopsy and immediate breast reconstruction with implant was performed. A histopathological report showed a massive high grade ductal carcinoma in situ, of the solid and comedo type. In one quadrant, some foci of microinvasions of less than 1 mm were present. Tumour margins were free. Isolated tumour cells were found in the sentinel lymph node. Hormone receptors were negative and human epidermal growth factor receptor-2 status was not performed. The patient received no adjuvant systemic therapy. Eight months after the surgery, she died from hepatic failure without known breast cancer progression before. An autopsy revealed diffuse liver metastases with human epidermal growth factor receptor 2-positive, hormone receptor negative breast cancer. Dissemination to other organs was not proven.

**Conclusion:**

Our patient is a rare case of ductal carcinoma in situ with microinvasion that developed distant metastases very early. In case of multiple foci of microinvasion, besides radical local treatment we suggest considering adjuvant systemic treatment based on biological characteristics since tumour size alone does not predict the prognosis well.

## Background

Microinvasive ductal carcinoma in situ (DCISM) is defined as a ductal carcinoma in situ (DCIS) of the breast with one or more foci of cancer cells that penetrate the basement membrane each measuring no more than 1 mm in the biggest dimension. Although it is considered as a subtype of DCIS, it is classified among invasive cancers as T1mic [1]. The crucial clinical-pathologic difference between DCIS and DCISM lies in its potential for metastasing. Recent reports on the histopathological findings, clinical presentations and imaging findings of DCISM have suggested that it may represent a distinct entity, with features different from those seen in pure DCIS [2,3]. However, its true metastatic potential is still unclear [2,3]. Mammographic findings of DCISM are not typical and could be absent. Similar to DCIS, the most frequent findings are microcalcifications. Mass, architectural distortions or asymmetry could also be found. Breast ultrasound may be helpful for additional examination [2]. Local treatment of DCISM is similar to the treatment of DCIS [4,5]. Obtaining clear surgical margins is the most important goal. Many women are amenable to breast-conserving surgery, whereas when an extensive and/or multifocal disease is present mastectomy is recommended. Immediate reconstructive surgery may be undertaken. Radiotherapy is indicated after breast-conserving surgery [4,5]. Opinions vary regarding the need for a sentinel node biopsy. In the past, the majority supported this procedure due to reports of positive sentinel nodes in up to 20% of cases [6]. In a recently published report, the rate of axillary lymph node metastases is low in patients with DCISM, it mainly concerns micrometastases or isolated tumour cells [7]. In the review of DCISM cases with negative axillary nodes the prognosis was excellent on the basis of surgical and radiotherapy-based local therapy only. Adjuvant hormonal therapy could be considered in hormone receptor positive tumours. In hormone receptor negative tumours with micrometastases in sentinel nodes, adjuvant chemotherapy could be considered, while in human epidermal growth factor receptor 2 (HER2) positive tumours chemotherapy and trastuzumab could be discussed [4]. We report a patient with a highly aggressive course of DCISM.

## Case presentation

**Figure 1:**
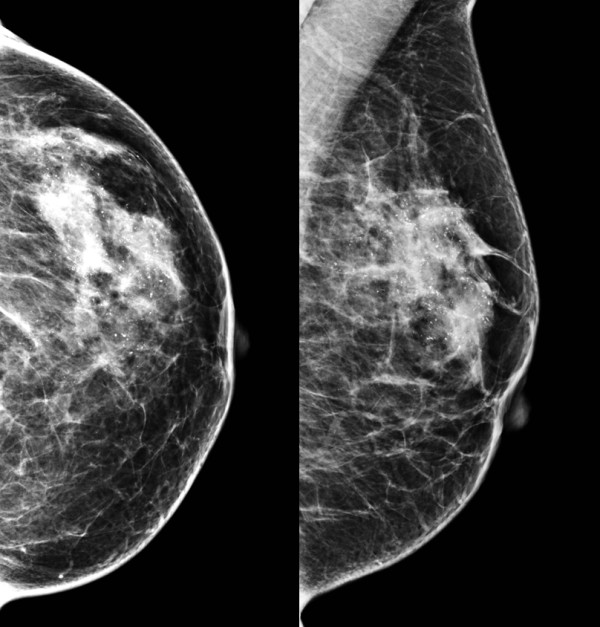
Mammographic appearance of ductal carcinoma in situ including microinvasive breast cancer.

**Figure 2:**
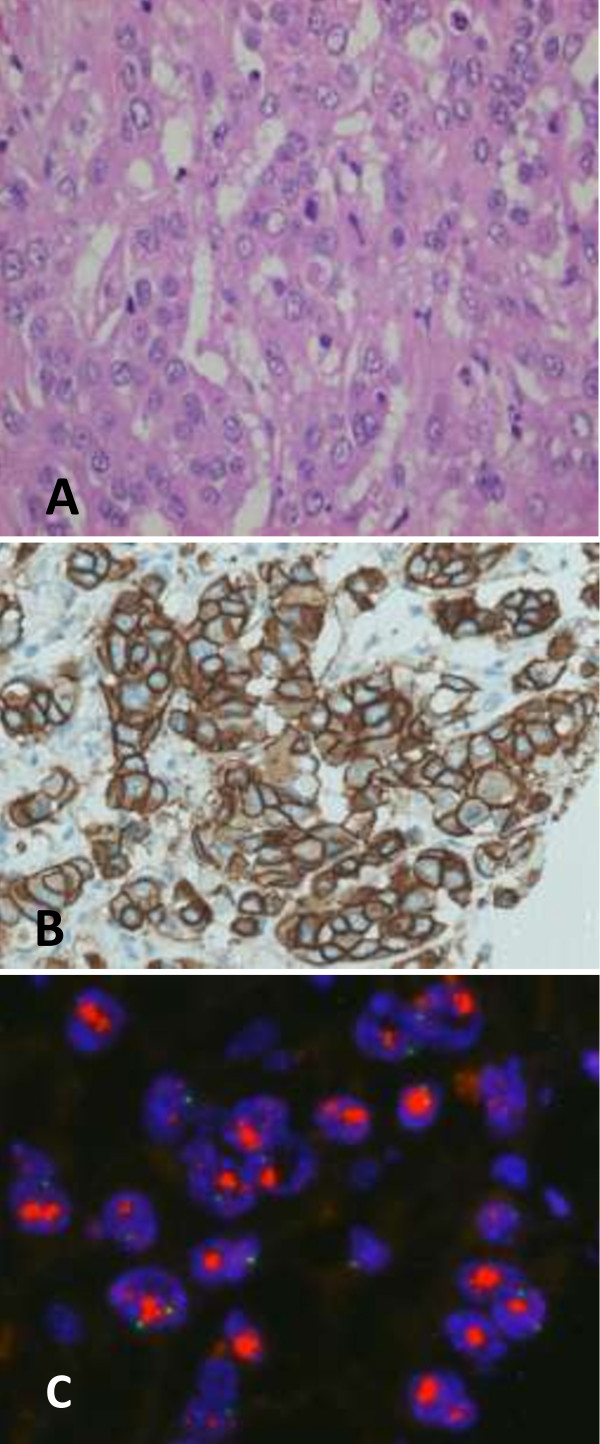
**Metastatic breast adenocarcinoma infiltrating liver. A**. Hematoxylin & eosin staining. **B**. Immunohistochemistry human epidermal growth factor receptor 2 oncoprotein staining: 3+ (PathWay® Human Epidermal Growth Factor Receptor 2/neu 4B5; Roche Inc.). **C**. Amplification of the Human Epidermal Growth Factor Receptor 2/neu gene: score > 10 (PathVysion® human epidermal growth factor receptor 2 Probe Kit; Abbott Molecular Inc.).

A 47-year-old premenopausal Caucasian woman presented in December 2009 during a multidisciplinary tumour board for nonpalpable lesions. Mammography revealed microcalcifications and gentle densities suspicious for invasion in the area of 6 × 8.6 cm in her left breast (Figure 1). An ultrasound showed indistinct hypoechoic lesions with posterior acoustic shadows. A core biopsy revealed high grade DCIS with calcifications. Due to the extensive microcalcifications, the patient was recommended to undergo a mastectomy and immediate breast reconstruction with expander implant. The surgery was performed on 23 March 2010. The mastectomy specimen weighed 238 g and measured 13 × 12 × 3 cm. Grossly, in the upper lateral quadrant of the breast a suspicious area was found. The suspicious focus measured around 2 cm in the largest diameter and consisted of somewhat harder yellow-whitish tissue of granular appearance. In the other parts, the breast tissue was mainly fatty without macroscopically suspicious changes. Careful sampling of the suspicious area and surrounding parenchyma was performed. Altogether, 12 tissue blocks were taken from that area. Besides, one paraffin block was taken from each quadrant and central part of the breast. Microscopic examination revealed massive, high grade DCIS with multiple foci of microinvasion that measured up to 0.1 mm in the largest diameter. Deeper sections from the paraffin blocks with microinvasion were also performed, but no invasive focus ≥1 mm was found. DCIS was located mainly in the upper lateral quadrant but multicentric foci of DCIS were present in all breast quadrants. The dimension of the DCIS was approximately 8 cm. Surgical margins were free (the basal margin was 6 mm). Tumour cells were negative for oestrogen (expressed in less than 1% of cells) and progesterone receptors (0%). Testing for HER2 status was not performed. In the pathologist’s opinion, the invasive clusters were too small for a reliable determination of HER2 status. Isolated tumour cells were found in the two sentinel lymph nodes. No adjuvant systemic therapy was recommended. The patient was regularly checked by a plastic surgeon from March to June 2010. In the meantime, filling of the expander implant with saline was performed. In June 2010 the patient went back to her work. On 23 September 2010, the replacement of the temporary implant for the permanent silicone prosthesis was performed. Blood count, electrolytes, urea and creatinine were in a normal range at that time. Laboratory liver function tests were not performed. She was examined for the last time by the plastic surgeon on 11 October 2010 when the sutures were removed and the wearing of an elastic bra was recommended. On 23 November 2010, the patient was referred to our institution after having been hospitalised for two weeks in a regional hospital due to the general deterioration of her health. Upon referral, she was icteric and in Eastern Cooperative Oncology Group performance status 3. Laboratory tests revealed leucocytosis, hypercalcaemia, deterioration of the liver and renal function and elevated tumour markers. Laboratory results upon referral were: leukocytes 14.8 × 10^9^/L, haemoglobin 121 g/L, platelets 167 × 10^9^/L, creatinine 145 μmol/L, urea 12 mmol/L, calcium 3.78 mmol/l, alkaline phosphatase 7 μkat/L, gama glutamil transferase 14 μkat/L, total bilirubin 95 μmol/L, direct bilirubin 84 μmol/L, aspartate transaminase 8.7 μkat/L, alanine transaminase 1.9 μkat/L, lactate dehydrogenase 7.9 μkat/L, albumins 26 g/L, C-reactive protein 69 mg/L, procalcitonin 4.1 μg/L, carcinoembryonic antigen 37000 μg/L, CA 15–3 432 kU/L and ammonium ion 187 μmol/L. An abdominal ultrasound was performed and showed ascites and an enlarged liver with diffuse lesions suspicious for metastases. A sample of ascites was sent for cytopathological examination. No malignant cells were found in the specimen. The hepatorenal failure progressed rapidly and the patient died on 29 November 2010. An autopsy was performed.In the autopsy a massive and diffuse infiltration of the liver with breast cancer metastases was found (Figure 2A). On immunohistochemical staining, tumour cells were negative for oestrogen and progesterone receptors and highly positive for HER2 (Figure 2B). The fluorescent in situ hybridization deoxyribonucleic acid probes score was over 10 (Figure 2C).

We report a highly aggressive course of DCISM in a premenopausal woman. She was treated with a mastectomy, sentinel lymph node biopsy and immediate breast reconstruction with implant. Tumour cells in microinvasive foci were hormone receptor negative and of unknown HER2 status. The sentinel lymph nodes showed no macrometastases but isolated tumour cells. The patient received no adjuvant systemic therapy. Eight months after the surgery she died from hepatic failure without her previously known breast cancer progressing. The autopsy revealed diffuse liver metastases with HER2-positive and hormone receptor negative breast cancer.

Despite the increasing incidence of DCIS as a consequence of widespread use of screening mammography, the incidence of DCISM remains low and accounts for around 1% of all breast cancers [2]. In general, the prognosis of DCISM is good and thought to be intermediate between pure DCIS and an early stage invasive cancer (T1a). Only a few reports present the local and distant recurrence rate and long-term outcome of patients with DCISM [2,3,7,8]. The heterogeneous definitions of microinvasion that were used in the past make clinical outcomes difficult to compare. In studies in which the current American Joint Committee on Cancer definition of microinvasion was used, the 5-year local relapse rate was between 0% and 3% and 5-year distant relapse free survival almost 100% [2,7,8]. The worst distant relapse free survival was reported by Parikh *et al*. In a group of 72 patients the 10-year distant relapse free survival was 97.9% [3].

We are currently still lacking a reliable test to accurately predict outcomes. Among prognostic factors for the outcome in DCISM, the number of microinvasive foci, metastases to the sentinel lymph node, high Ki-67, negative oestrogen receptor status, and HER2 overexpression were found [3,9]. The number of microinvasive foci is probably higher in more extensive DCIS. The size and extent of DCIS lesions are often only an estimate [2]. Quantifying the size of a DCIS lesion is difficult and precise measurement is often impossible. Still, an estimation of the DCIS size is clinically very important. According to the College of American Pathologists guidelines for examining specimens from patients with DCIS, microscopic examination of the entire area affected by DCIS is recommended but is very often impractical if DCIS is larger than 4 (even 2) cm [10]. In such a case, there is always a possibility of undetected areas of invasion if the area affected by DCIS is not completely examined. As well, smaller invasive foci could be missed because they can be present deeper in that part of the paraffin block that was not histologically examined. It is difficult for the pathologist to find all possible foci of microinvasion in very extensive DCIS. The multifocality of microinvasive disease is in correlation with positive sentinel nodes [6,11].

In invasive breast cancer, axillary lymph node metastases are the most powerful prognostic factor for relapse and survival. The risk of sentinel lymph node metastases among patients with DCISM has been reported to be between 0 and 20% [6]. It is crucial to identify a very small subset of microinvasive cancer patients with macrometastases, which could benefit from adjuvant systemic therapy [7]. In case of a positive sentinel node, an axillary dissection should be performed [2,6,7]. In case of positive axillary nodes, adjuvant systemic therapy with tamoxifen in hormone receptor positive tumours, and with chemotherapy in hormone receptor negative tumours could be offered. In invasive breast cancer, micrometastases but not isolated tumour cells were associated with additional positive nodes upon axillary dissection and with distant recurrence [11]. Most authors agree that a sentinel node biopsy should be performed although some believe it could be omitted due to a low rate of positivity [7,8]. The outcome is generally very good with few local and even fewer distant relapses [2,3]. At present, the significance of lymph node micro- or macrometastatic disease on overall survival in DCISM is unclear and needs to be ascertained with further studies. Colleoni *et al.* reported increased Ki-67 as a significant prognostic factor for disease free survival in DCISM [12].

HER2 overexpression was present in 77% of locally relapsed tumours after breast-conserving therapy for DCIS (with or without radiation therapy in European Organisation for Research and Treatment of Cancer trial 10853) [13]. A clinical implication of HER2 status in DCIS is unclear. On the contrary, HER2 overexpression in even small invasive cancers is correlated with poor prognosis. Roses *et al.* found HER2 status to be a predictor for the transition from in situ to invasive breast cancer [14]. However, there are some technical constraints on HER2 testing as a certain volume/number of malignant cells in a cluster should be found for reliable testing. One of the on-going trials in HER2-positive DCIS involves the testing of a single application of neoadjuvant trastuzumab in <1 cm DCIS before surgery. The second trial is a phase III National Surgical Adjuvant Breast and Bowel Project B43 trial comparing adjuvant radiotherapy with or without two doses of adjuvant trastuzumab in HER2-positive DCIS after breast-conserving surgery [15]. There are also promising results of clinical studies with HER2-pulsed autologous dendritic cell vaccine administered before surgical resection of high-risk HER2-positive DCIS [16,17]. The results of those trials will hopefully show more insights into the biology of HER2-positive DCIS and DCISM.

Mori *et al.* evaluated almost 400 tumour specimens of patients who were diagnosed with either DCIS, DCISM or T1a breast cancer. The comedo type and ER negative/HER2-positive type were found more frequently in DCISM than in either DCIS or T1a tumours. The co-expression of HER2 and 14.3.3 zeta could be important since HER2 overexpression plays a role in the process of first invasion and 14.3.3. zeta in the reduction of cell adhesion [18].

Our patient had many potential risk factors for relapse. She had a very extensive DCIS in the whole breast with more than one microinvasive focus within DCIS. DCIS was of a comedo subtype. She had no micro- or macrometastases but isolated tumour cells were present in the sentinel lymph node, which suggested a more aggressive disease. Tumour cells were negative for oestrogen and progesterone receptors. HER2 status was not determined due to an unreliable result determined on a scant sample. The patient received no adjuvant systemic therapy. The number of invasive foci and the extent of DCIS could be very suspicious of many microinvasive foci. In that specific case, we did not consult a pathologist for the additional counting of invasive foci. Following this case, the general practice at our institution has changed. If a pathologist finds more than 5 microinvasive foci (where the sum of the sizes is at least 5 mm) we discuss adjuvant chemotherapy with the patient.

## Conclusion

Like DCIS, DCISM is a disease with a favourable prognosis. However, there are rare cases that relapse either locally or distantly. At present, we are unable to identify those at a higher risk of relapse. HER2 overexpression probably plays an important role. The results of the on-going studies will hopefully help us better understand the nature of this disease and guide us to select patients with an aggressive subtype of DCIS and DCISM that would benefit from additional systemic therapy.

We conclude that in a massive DCISM, besides precise anatomical staging, hormone receptor and also HER2 status has to be determined. In cases with HER2-positive and hormone receptor negative tumours adjuvant systemic therapy should be considered or a clinical trial offered, especially for those with micrometastases or isolated tumour cells in the sentinel lymph node.

## Consent

“Written informed consent was obtained from the patient’s next of kin for publication of this case report and accompanying images. A copy of the written consent is available for review by the Editor-in-Chief of this journal.”

## Abbreviations

DCIS: Ductal carcinoma in situ; DCISM: Microinvasive ductal carcinoma in situ; HER2: Human Epidermal Growth Factor Receptor 2.

## Competing interests

The authors declare that they have no competing interests.

## Authors’ contributions

CGK analysed and interpreted the patient data and was a major contributor in writing the manuscript. EM helped interpret the patient data and write the discussion. Both authors read and approved the final manuscript.
